# Breast composition during and after puberty: the Chilean Growth and Obesity Cohort Study

**DOI:** 10.1186/s13058-024-01793-x

**Published:** 2024-03-12

**Authors:** Ana Pereira, María Luisa Garmendia, Valeria Leiva, Camila Corvalán, Karin B. Michels, John Shepherd

**Affiliations:** 1 Institute of Nutrition and Food Technology, University of Chile, Santiago, Chile; 2 MRC Biostatistics Unit, University of Cambridge, Cambridge, UK; 3 Department of Epidemiology, Fielding School of Public Health, University of California, Los Angeles, USA; 4 Institute for Prevention and Cancer Epidemiology, Faculty of Medicine and Medical Center, University of Freiburg, Freiburg, Germany; 5 Population Sciences in the Pacific Program (Cancer Epidemiology), University of Hawaii Cancer Center, Honolulu, HI USA

**Keywords:** Breast composition, Puberty, Latino girls, Cohort study

## Abstract

**Background:**

Breast density (BD) is a strong risk factor for breast cancer. Little is known about how BD develops during puberty. Understanding BD trajectories during puberty and its determinants could be crucial for promoting preventive actions against breast cancer (BC) at early ages. The objective of this research is to characterize % fibroglandular volume (%FGV), absolute fibroglandular volume (AFGV), and breast volume (BV) at different breast Tanner stages until 4-year post menarche in a Latino cohort and to assess determinants of high %FGV and AFGV during puberty and in a fully mature breast.

**Methods:**

This is a longitudinal follow-up of 509 girls from low-middle socioeconomic status of the Southeast area of Santiago, recruited at a mean age of 3.5 years. The inclusion criteria were singleton birth born, birthweight between 2500 and 4500 g with no medical or mental disorder. A trained dietitian measured weight and height since 3.5 years old and sexual maturation from 8 years old (breast Tanner stages and age at menarche onset). Using standardized methods, BD was measured using dual-energy X-ray absorptiometry (DXA) in various developmental periods (breast Tanner stage B1 until 4 years after menarche onset).

**Results:**

In the 509 girls, we collected 1,442 breast DXA scans; the mean age at Tanner B4 was 11.3 years. %FGV increased across breast Tanner stages and peaked 250 days after menarche. AFGV and BV peaked 2 years after menarche onset. Girls in the highest quartiles of %FGV, AFGV, and BV at Tanner B4 and B5 before menarche onset had the highest values thereafter until 4 years after menarche onset. The most important determinants of %FGV and AFGV variability were BMI z-score (R^2^ = 44%) and time since menarche (R^2^ = 42%), respectively.

**Conclusion:**

We characterize the breast development during puberty, a critical window of susceptibility. Although the onset of menarche is a key milestone for breast development, we observed that girls in the highest quartiles of %FGV and AFGV tracked in that group afterwards. Following these participants in adulthood would be of interest to understand the changes in breast composition during this period and its potential link with BC risk.

**Supplementary Information:**

The online version contains supplementary material available at 10.1186/s13058-024-01793-x.

## Background

Breast cancer (BC) is the most common female cancer globally [1], with many risk factors developing early in life [2]. Birth weight, birth height, and adolescent height are all positively associated, while gestational age and higher body mass index (BMI) at 18 to 30 years are inversely associated with BC risk [3]. Breast tissue develops under the influence of ovarian hormones (estrogen and progesterone) in puberty, but it does not fully differentiate until after the first full-term pregnancy [4]. Thus, early life is a susceptible period for the effects of carcinogens on the breast gland [5]. However, it is not easy to assess these relationships prospectively because of the long time between exposures and BC.

The percentage of dense breast tissue is one of the strongest risk factors for BC [6, 7]. Breast density (BD) is highly heritable and a large proportion of variation in breast density across women can be explained by genetic factors, with heritability estimates around 0.6 as seen in monozygotic and dizygotic twins’ studies [8]. A recent cohort study observed that premenopausal women with a family history of BC were more likely to have higher BD [9]. Breast density is achieved early in life and decreases with age [10]. There is little work to date on how BD develops, and its determinants in early life since BD is typically measured from mammograms. Due to radiation exposure, mammograms are not feasible in adolescents and young women. DXA is an alternative technique for measuring BD; it has a low radiation dose (approximately 1 day of background radiation), is inexpensive, does not require breast compression, is operator independent, and provides a 3-dimensional calibrated image [11]. In 2002, Shepherd and colleagues observed that DXA % of fibroglandular volume (%FGV) strongly correlates with BD measured from mammograms [11, 12]. Furthermore, a study using duplicate DXA breast scans on the same day in 200 pre-menopausal women observed a test-retest precision of 2.8% [12]. A cross-sectional study using DXA in pubertal girls observed that %FGV peaked at Tanner Breast stage 4 [13, 14]. After that, the absolute amount of fibroglandular tissue (AFGV) remains stable while non-dense breast tissue (adipose tissue) increased, resulting in a decrease of %FGV [13, 14]. It is unknown if girls with high BD at or near puberty remain high after puberty. In this study, we recruited girls from a well-characterized birth cohort before they had transitioned through puberty to (i) estimate longitudinally the time when the peak of BD occurs and if high BD persists well after menarche (ii) to assess longitudinally the determinants of high BD during puberty, and (iii) to assess early determinants of BD at the end of puberty (in a fully mature breast, 4 years after menarche onset) in the Chilean population.

## Methods

### Study design and population

The Growth and Obesity Chilean Cohort Study (GOCS) started in 2006; details of the GOCS study and methods have been extensively published elsewhere [15]. Briefly, 1190 children (602 girls) from low-medium socioeconomic level were recruited from the South-East area of Santiago, Chile. The girls were included if they were born in 2002–2003, had a single-term birth, birthweight of 2500gr-4500grs, and were free from mental or physical abnormalities.

Measures relevant to this study include annual anthropometric (since 2006, mean age 3.5 years), maternal mammographic BD, sexual maturation assessment, and biennial Tanner staging (since 2010, mean age 8 years). From the 602 recruited girls, sexual maturation was assessed in 509 girls; ninety-three girls abandoned the study before 2010, when we started measuring breast tanner stages systematically (we could not contact 24 girls anymore, and 69 abandoned the study voluntarily between 2006 and 2010). The excluded girls (*n* = 93) were not different from included girls in terms of birth weight, BMI z-score, height z-score, and weight z-score at recruitment and maternal education (supplemental Table 1).

Specific for this study, DXA breast scans were performed randomly in 7 girls at Tanner B1, 11 girls at Tanner B2, 10 girls at Tanner B3, 388 girls at Tanner B4, 183 girls at Tanner B5, 144 girls at 1-year after menarche, 326 girls at 2-year after menarche and 373 at 4-year after menarche. Thus, during 2012–2019, we carried out 1,442 breast DXA scans in the 509 girls which 442 had at least two breast DXA exams.

In 2010–2011, we also evaluated breast composition in the girls’ mothers (*N* = 353).

### Measurements

All participating girls were evaluated at the outpatient clinic of the Institute of Nutrition and Food Technology at the University of Chile in Santiago, Chile, from 2012 to 2019.

#### Pubertal assessment

A female dietitian trained by a pediatric endocrinologist performed breast evaluations using Tanner’s scale [16, 17] in a private room in the presence of the mother or other adult; it included visual inspection and palpation of the girl’s breast [18]. Menarche onset was assessed at every visit to the Institute of Nutrition and Food Technology (INTA), and after Tanner B4, they were followed by phone every three months.

#### Anthropometric measures

Trained and standardized dietitians measured the weight and height of the participants dressed in light clothing and barefooted using a Tanita 418 BC (accuracy of 0.1 kg) and a stadiometer Seca 222 (accuracy of 1 mm), respectively. We estimated the BMI as weight/height^2^ (kg/m^2^) and calculated the z-score for height and BMI based on World Health Organization (WHO) 2007 standards [19].

#### Breast composition data

In the daughters, breast composition was measured using a DXA from GE Lunar Prodigy Bone Densitometer (GE Healthcare, Madison, WI, USA) calibrated to measure AFGV, BV, and %FGV using a custom thickness step phantom [20]. The phantom is made of reference material which consists of seven density steps with different percentages of breast fibroglandular (from 0 to 100%) and adipose tissue [21, 22]. Details of the technique have been published elsewhere [11, 23]. Briefly, a single trained technician performed DXA measurements. All participants were dressed in a loose light gown covering the chest area, and breast scans were taken with the participant in a lateral decubitus position. We first scanned the left breast, followed by the right breast. The expected radiation exposure in the DXA breast scan is 0.15 mSV; 10 times lower than a mammogram [13]. DXA breast scans were read using a software developed at the University of California San Francisco (co-author JS [11]). Following a standard protocol, a single trained reader (intra and inter-rater correlation coefficient > 0.9) manually delineated the total projected breast area, and the software automatically estimated %FGV, AFGV, and BV.

In the mothers, we performed a digital mammography (Hologic Selenia, Marlborough, MA, USA) of both breasts in a private clinic of Santiago during the first week of their menstrual cycle. The %FGV and AFGV were estimated in the raw images using VOLPARA software (version 1.4.2, Matakina, Technology, Wellington, New Zealand).

### Statistical analysis

We described the study population (mean and standard deviation (SD)) in terms of age at DXA, weight, BMI, and height z-scores at Tanner B4. We plotted the median of %FGV, AFGV, and BV at Tanner 1, 2, 3, 4, and 5 (the last two stages (B4 and B5) were classified according to menarche status at the time of DXA measurement) and 1, 2, and 4-year after menarche onset. Usually, menarche arrives when girls transition between Tanner B4 and B5; however, some girls reach first Tanner B5 and then menarche. Thus, we also classified DXA scans at Tanner B4 and B5 according to menarche status. Girls could have had their DXA scan at B4 or B5 before the menarche onset or their DXA scan at B4 or B5 after menarche onset but within the first six months since menarche; if not, they were classified as 1y after menarche onset. We tested statistical differences among groups using the Mann-Whitney test.

We graphed the association between %FGV, AFGV, and BV and the number of days (before and after) of menarche onset and to chronological age. We used the ns function from R to estimate the natural spline regression of each of the parameters, and we defined the knots in which the slope of the curve changed (i.e., the relationship between %FGV, AFGV, and BV and days from menarche change and chronological age). The knots selected for each curve were domain-specific, in other words, defined visually by the researchers, and they were cross-validated.

We collapsed and stratified the data in 4 times points: (i) Tanner B4 or B5 before menarche, (ii) Tanner B4 or B5 after menarche, (iii) 2 years, and (iv) 4 years after menarche. Using Tanner B4 or B5 data before menarche, we categorized the breast composition in quartiles according to its distribution. Following this procedure, we graphed the mean value of breast composition data in time to assess if the group with the highest values remained highest in the following periods. We further estimated the mother’s %FGV across the quartiles of the girl’s %FGV and performed a test for trend across ordered groups to assess the correlation between mothers’ and daughters’ %FGV.

We performed linear mixed-effect models assuming random intercept and slope to assess which biological maturation and/or nutritional status determinants were associated with %FGV and AFGV during puberty. We estimated the coefficient of determination (R^2^) proposed by Snijders and Bosker [24] that considers girls could have more than one DXA (DXA measurements within girls are not independent). The aim was to assess which of these biological or nutritional markers better explained breast composition variability during puberty.

Finally, we wanted to build a multivariable model to better explain the %FGV and AFGV in a fully mature breast (4 years after menarche onset) for Chilean population. For this, we performed a backward stepwise linear regression model assuming a probability for variable removal of 0.1.

### Ethics

The Institutional Review Board of the Institute of Nutrition and Food Technology, University of Chile, has approved the GOCS studies. All girls and their responsible adult care-taker agreed to participate by signing an assent and informed consent, respectively.

## Results

Table 1 describes the participant’s general characteristics at Tanner B4; 15% of the girls were obese at Tanner B4 (mean age = 11.3 years, SD = 0.9), and mothers had a mean age of 35 years (SD = 6.6) (data not shown in Table).

**Table 1:** Characteristics of girls at different maturational stages. The Growth and Obesity Cohort Study

	Tanner B4 *n* = 388	Tanner B5 *n* = 183	1 year after menarche *n* = 144	2 years after menarche *n* = 369	4 years after menarche *n* = 373
	mean (sd)	mean (sd)	mean (sd)	mean (sd)	mean (sd)
**Age (years)**	11.3(0.8)	11.7(0.7)	12.6 (1.2)	14.0 (1.1)	16.0 (0.8)
**Weight (kgs)**	45.7 (8.8)	51.0 (10.0)	56.0 (10.5)	59.0 (12.9)	61.9 (12.4)
**Height (cms)**	149.5 (6.7)	153.6 (5.4)	156.5 (6.3)	158.2 (6.1)	159.4 (5.8)
**BMI (kgs/mt2)**	20.4 (3.2)	21.9 (3.8)	22.8 (3.8)	23.5 (4.6)	24.3 (4.4)
**Height z-score**	0.4(1.0)	0.5(0.9)	0.4(0.9)	-0.1(0.9)	-0.4 (0.8)
**BMI z-score**	0.9(1.0)	1.2(1.1)	1.2(1.0)	1.0(1.1)	0.9 (1.0)
**Nutritional Status (n,%)**					
Normal (1sd)	226 (50.0)	76 (38.8)	58 (40.3)	158 (48.6)	202 (55.5)
Overweight (1-2sd)	162 (35.8)	74 (37.8)	80 (34.7)	109 (33.5)	106 (29.1)
Obese (> 2sd)	64 (14.2)	46 (23.5)	36 (25.0)	58 (17.9)	56 (15.4)
**%FGV**	42.7 (16.1)	45.0 (15.8)	49.9 (14.3)	50.0 (14.9)	49.5 (15.6)
**AFGV**	91.3 (40.3)	133.5 (54.2)	186.6 (71.5)	215.8 (76.4)	223.1 (82.9)
**BV**	231.5 (103.3)	318.7 (139.6)	400.6 (178.7)	460.5 (198.4)	491.0 (226.6)

BMI: body mass index

* nutritional status in girls was according to WHO SDS (normal < 1SD, overweight 1SD-2SD, obese > 2SD), in mothers according to

%FGV: % fibroglandular volume; AFGV: absolute fibroglandular volume; BV: breast volume

**Fig. 1:**
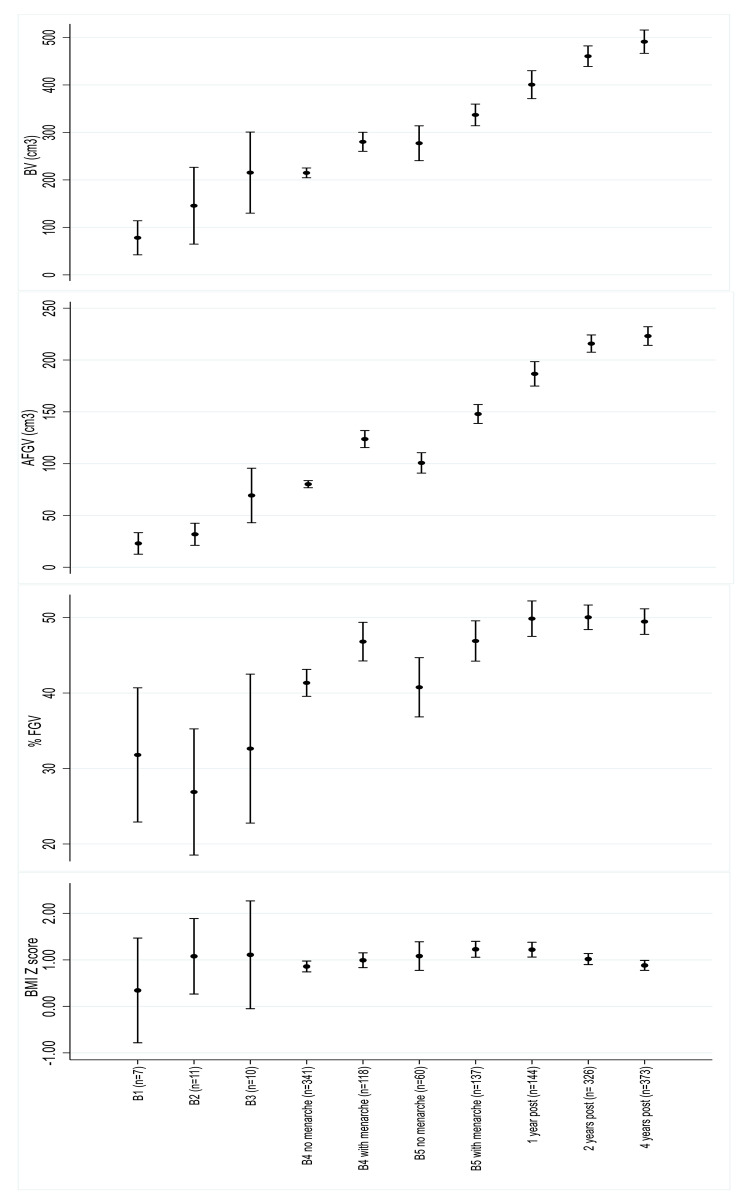
**Description of breast composition and BMI z score in girls at different maturation ages**. **The Growth and Obesity Cohort Study (n=1,442 breast DXA scans).** The figure represents the mean and standard deviation of breast composition components (%FGV, AFGV, and BV) and BMI at different time points of maturation: Tanner stage breast (B) B1, B2, B3, B4 before menarche onset, B4 after menarche onset, B5 before menarche onset, B5 after menarche onset, 1, 2 and 4 years after menarche onset. BV: breast volume; AFGV: absolute fibroglandular volume; %FGV: % of fibroglandular volume; BMI z-score: body mass index z-scores according to World Health Organization data 2007

Figure 1 shows that AFGV and BV increase across Tanner B1 to 2 years post menarche onset; statistical differences within periods are shown in Supplemental Table 2. %FGV is determined by menarche onset with %FGV increasing until 1y after menarche and thereafter remaining stable (Tanner B4 after menarche (mean = 46.8%, SD = 13.8%), Tanner B5 after menarche (mean = 46.9% and SD = 15.8%), 1-year after menarche (mean = 49.9%, SD = 14.3%) and 2- year after menarche (mean = 50.0%, SD = 14.9%)).

**Fig. 2:**
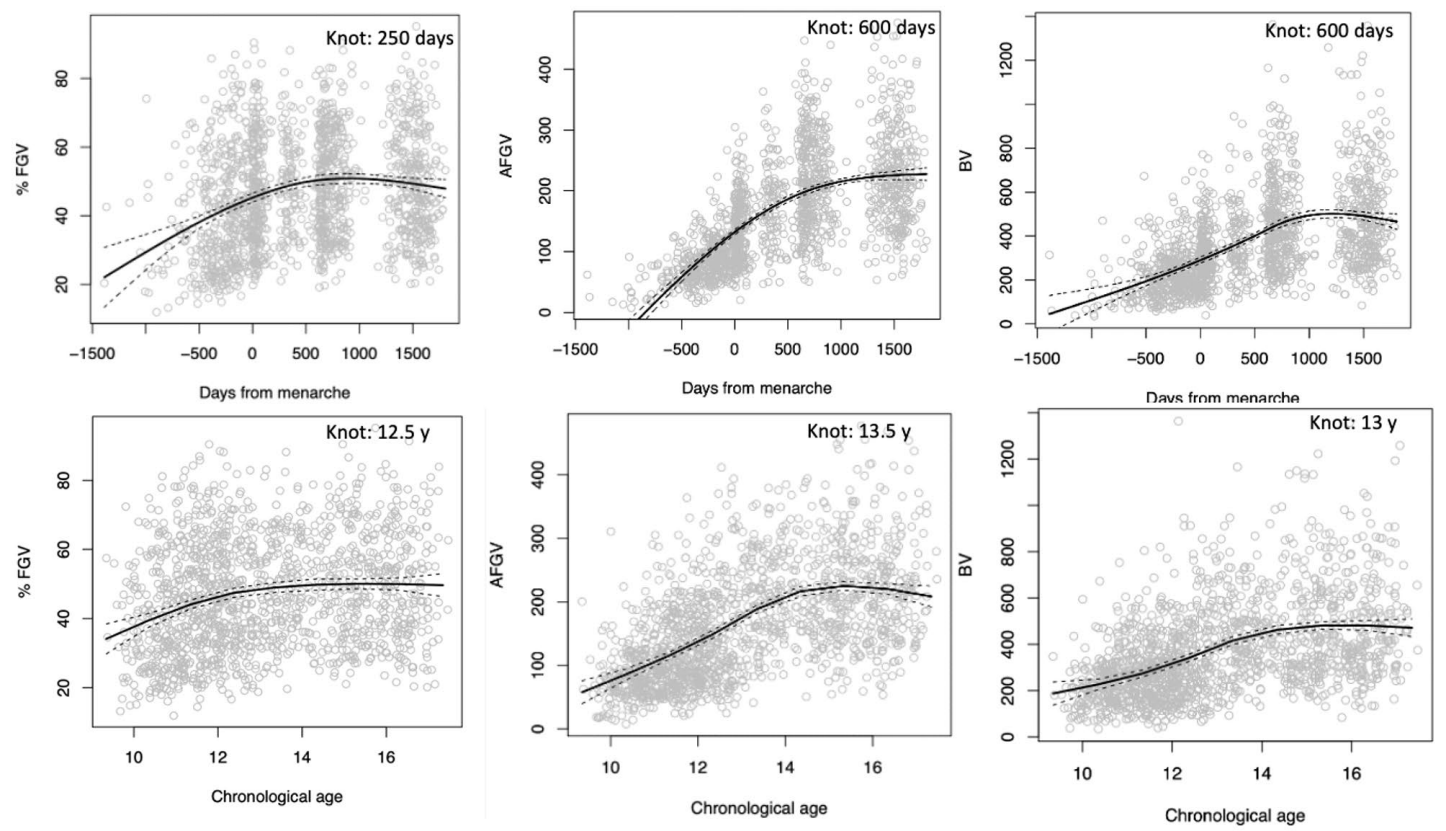
**Distribution of Breast composition as function of days from onset of menarche and chronological age.** **The Growth and Obesity Cohort Study (n=1,442 breast DXA scans).** %FGV: %fibroglandular volume; AFGV: absolute fibroglandular volume; BV: breast volume

Figure 2 depicts %FGV, AFGV, and BV in relation to time to menarche onset (0 corresponds to the day of menarche onset) and chronological age. Using visual observation of the curve, we observed a knot at 250 days after menarche onset for %FGV; the relationship is linearly significant up to 250 days from menarche onset, but after 250 days, the relation becomes non-significant. In parallel, we observed a knot in %FGV at age 12.5 years. For AFGV and BV, we find a knot 600 days after menarche onset and at 13.5 and 13 years, respectively.

**Fig. 3:**
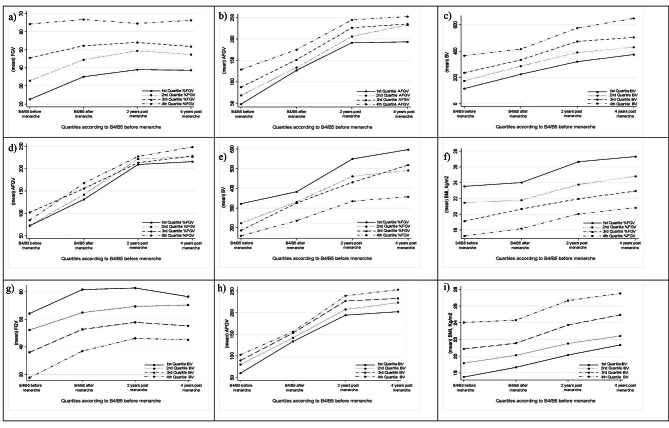
**Distribution of breast composition (%FGV, AFGV, BV) and BMI across puberty. The Growth and Obesity Cohort Study (n=1,442 breast DXA scans).** %FGV: %fibroglandular volumen; AFGV: absolute fibroglandular volume; BV: breast volume. **a**) data is stratified according to quartiles of %FGV at Tanner B4 or B5 before menarche onset, **b**) data is stratified according to quartiles of AFGV at Tanner B4 or B5 before menarche onset, **c**) data is stratified according to quartiles of BV at Tanner B4 or B5 before menarche onset, **d**) and **e**) data is stratified and followed according to quartiles of %FGV at Tanner B4 and B5 before menarche onset, **f**) data is stratified according to quartiles of BMI at Tanner B4 or B5 before menarche onset, **g**) **h**) and **i**) data is stratified according to quartiles of BV B4 or B5 before menarche onset

In Fig. 3a, b, c, and f, we observed that girls in the highest quartile of %FGV, AFGV, BV, or BMI at Tanner B4 or B5 before menarche onset are in the highest quartile of distribution in the subsequent visits. However, data in AFGV is less clear when we stratify the data according to %FGV distribution at Tanner B4 and Tanner B5 before menarche onset (Fig. 3d). Instead, girls in the highest quartile of %FGV at Tanner B4 and Tanner B5 before menarche onset have the highest BV across puberty (Fig. 3e). We observed that stratifying by BV before menarche onset, girls in the first quartile had the highest %FGV (Fig. 3g), but lowest AFGV and BMI during the following four time periods (Fig. 3h and i). After stratifying by the daughters’ %FGV at Tanner B4 and Tanner B5 before menarche, we observed that mothers’ %FGV in the 1st quartile was 9.5% (SD = 4.8), in the 2nd quartile 9.6% (SD = 4.9), in the 3rd 10.9% (SD = 6) and in the 4th 11.0% (SD = 5.6) (Test for trend across ordered groups, *p*-value = 0.037) (data not shown in table).

In Table 2, the crude and adjusted models shows that BMI z-score is the most important variable to explain %FGV variability during puberty, with an R^2^ of 44%. Tanner staging, menarche status, time to menarche, age, height and mother’s %FGV were also associated with %FGV but each explained less than 4% of the variation. All girl’s variables were positively related to AFGV during puberty (mother’s AFGV had a borderline significance), but time to menarche to DXA was the variable that explained the most variability of AFGV (39%) according to R^2^; on the other hand the BMI z-score explained only 2% of the variability.

**Table 2:** Determinants of breast composition during puberty. Crude and adjusted mixed-effect models. The Growth and Obesity Cohort Study (*n* = 1,442 breast DXA scans)

	Model 1: Crude Models				Model 2: Each variable adjusted by age and BMI				
	β	95% CI	*p*-value	R^2^	β		95% CI	*p*-value	R^2^
	% Fibroglandular Volume								
Tanner stage	6.04	4.35 ; 7.73	< 0.001	0.01	6.33		4.63 ; 8.03	< 0.001	0.47
Menarche at DXA (yes/no)	8.04	6.79 ; 9.29	< 0.001	0.01	9.74		8.31 ; 11.17	< 0.001	0.53
Time to menarche (days)	0.01	0.004 ; 0.006	< 0.001	0.04	0.01		0.01 ; 0.02	< 0.001	0.50
Age (years)	1.85	1.60 ; 2.11	< 0.001	0.03	1.62*		1.39 ; 1.85	< 0.001	0.44
BMI z-score at DXA	-10.17	-10.92 ; -9.43	< 0.001	0.44	-9.80#		-10.54 ; -9.06	< 0.001	0.44
Height z-score at DXA	-2.64	-3.55 ; -1.72	< 0.001	0.003	0.26		0.03 ; 0.49	0.027	0.44
Mother´s %FGV	0.46	0.17; 0.75	0.002	0.02	0.18		-0.03; 0.39	0.085	0.44
	Absolute Fibroglandular Volume								
Tanner stage	53.03	45.88 ; 60.18	< 0.001	0.25	30.60		23.02 ; 38.18	< 0.001	0.32
Menarche at DXA (yes/no)	55.90	50.06 ; 61.75	< 0.001	0.29	50.16		43.66 ; 56.66	< 0.001	0.30
Time to menarche (days)	0.08	0.08 ; 0.08	< 0.001	0.39	0.07		0.05 ; 0.08	< 0.001	0.42
Age (years)	25.69	24.01 ; 27.37	< 0.001	0.31	26.63*		24.98 ; 28.28	< 0.001	0.36
BMI z-score at DXA	6.66	2.46 ; 10.85	0.002	0.02	16.19#		12.56 ; 19.83	< 0.001	0.36
Height z-score at DXA	-8.06	-12.37 ; -3.76	< 0.001	0.02	2.69		-1.95 ; 7.34	0.256	0.37
Mother’s AFGV	-0.07	-0.27; 0.14	0.536	0.02	0.18	0.17	-0.004; 0.34	0.056	0.37

* adjusted only by BMI z-score

# adjusted only by age at DXA in years

β: beta coefficient, 95%CI: 95% confidence interval, R^2^: coefficient of determination

DXA: Dual-energy X-Ray Absorptiometry, %FGV: % of fibroglandular volume, AFGV: absolute fibroglandular volume; BV: breast volume. BMI: body mass index

We further estimated the best predictor model of %FGV and AFGV in a fully mature breast 4 years after menarche onset (Supplemental Table 3). In the case of %FGV, the variables selected in the multivariable model were BMI z-score at 4 years after menarche onset and age, and for AFGV the variables were BMI z-score and mother’s AFGV.

## Discussion

To our knowledge, this is the first study assessing breast composition from puberty until 4 years after menarche onset. Menarche onset is a key developmental event for the acquisition of %FGV, a proxy of BD. %FGV increases during puberty until 1 year after menarche onset; then, it remains constant. Conversely, AFGV and BV continue increasing until 2 years after menarche. Also, we observed that girls that tracked early (before menarche onset) in the group of highest %FGV, AFGV, and BV, remained in the highest group in the following years. As expected, the main determinant to explain %FGV variability during puberty in girls was BMI z-score; however, the primary determinant in this period for the AFGV was the number of days before or after menarche onset of the DXA was taken. Furthermore, we built a multivariable model to estimate the %FGV and AFGV in a fully mature breast (4 years after menarche onset) using biological and nutritional determinants, which could allow us to characterize girls at higher risk, that could be applicable for Chilean population.

Few studies have assessed breast composition during and after puberty. The first study assessed DXA breast composition in 18 girls between 13 and 14 years of age. These authors observed that %FGV was nearly 100% between Tanner B1 to Tanner B4, achieving an AFGV of approximately 200 cm^3^ at B4. At Tanner B5, they observed a large increase in BV, thus decreasing %FGV [14]. The increase of subcutaneous adipose tissue between Tanner B4 and Tanner B5 was also described using ultrasound [25]. Later, a larger cross-sectional study assessed 113 girls (10.2 to 16.9y) from different ethnic groups of Hawaii [13] and showed different data compared to our results. Hawaiian girls at Tanner stage 4 were older and had higher %FGV, BV, and BMI than our participants (%FGV: 70.9% vs. 42.7%; BV 324 vs. 227.8 cm^3^ and BMI: 21.0 vs. 20.4 kg/m^2^, respectively). They observed that the highest peak of %FGV was at Tanner B4 and that AFGV and BV increased until Tanner 5. In contrast, we report that %FGV peaks and remains stable after 8 to 9 months of the menarche onset. However, our participants’ peak %FGV was at 12.5 years, a lower age than the Hawaiian girls at B4 (14.3 years). They also observed that body fatness explained 69% of the variability in %FGV and menarche onset only 9%. In our case, body fatness explained 44% and menarche onset 1% of %FGV variability. Instead, menarche mainly explained AFGV variability (39%), but BMI z-score only explained 2%. The Dietary Intervention Study in Children (DISC) performed magnetic resonance imaging in 174 women aged between 25 and 29 years, and they observed an inverse association between body fatness and %FGV and AFGV [26], while we observed an inverse association with %FGV and positive with AFGV, similar to a recent study published by Lloyd 2023 in 169 women aged between 18 and 40 year [27] and our previous findings in premenopausal women [12]. Most of the studies carried out in pre or post-menopausal women agree with the inverse association between body fatness and %FGV, but the evidence with AFGV remains controversial [12, 28–30]. The breast contains deposits of fat tissue, and a recent study concluded that an increase in the BMI increases the breast volume and breast-dense tissue but reduces the % of BD, but the association between BMI and dense tissue is less strong than the inverse association with % BD [31].

Additionally, since the breast is a deposit for adipose tissue, high attained BMI is strongly associated with high levels of breast fat area [32–35] and breast fat volume [36, 37], which in turn leads to an inverse association between BMI and both percent dense area [32–35, 38, 39] and percent dense volume [36, 37, 40–42].

Our study shows that menarche is pivotal to determining %FGV and AFGV. Girls are born with a rudimentary mammary gland structure [43, 44]. During puberty and until the first birth, the mammary gland starts to divide, and terminal end buds and rudimentary alveolar are created (lobules type 1 and type 2), [36]. These lobules have higher cell replication rates, making this period more sensitive to external and internal factors that may lead to mutations. Earlier age at menarche onset is an important risk factor for pre-and post-menopausal BC [37, 40], as well as a longer time interval between menarche onset and the first full-term pregnancy [32]. In contrast, the association between menarche onset and BD is less consistent. Several studies have shown no association [33, 34], while other studies have observed a protective effect [35]. The International Consortium of Mammographic Density, a pooled analysis of 10,681 breast-cancer‐free women from 22 countries, reported that women with later age at menarche onset had higher percent mammographic density and absolute dense area (mean age at mammogram was 52.7 years (sd = 8.2y)) [38]. On the other hand, the DISC study reported that girls with earlier thelarche onset or a longer pubertal tempo between thelarche and menarche onset had higher %FGV at age 25–29 years [39]. However, none of these studies assessed the relationship between menarche onset and BD at the beginning of reproductive life. Interestingly, we observed that puberty onset is a key determinant for %FGV and AFGV during puberty but not for a fully mature breast at 4 years after menarche onset. Earlier menarche onset may be linked to increased BC risk through the earlier acquisition of lobules 1 and 2 [5] and, therefore, an extension of the susceptible period for carcinogenic effect. Our results showed that AFGV starts increasing after Tanner 2 until 2 years after menarche. We could expect that a longer pubertal tempo would increase AFGV and potentially BC in adult life [41]. Moreover, our data suggest that %FGV and AFGV tracks during and after pubertal onset; girls that belonged to the highest quartile of %FGV and AFGV before menarche remained in the same group in the following visits. Boyd et al. in 2009, using data from mother and daughter pairs, postulated that BD variability was highest at early ages. Women in the highest group would have a higher decrease in BD during life-course than women with lower BD but still will have higher BD with increasing age [10].

In our study, we found a positive correlation between the mother’s and daughter’s BD (the mothers of the daughters in the highest quartile of %FGV, also had the highest %BD), and the mother’s BD predicted the girl’s %FGV and AFGV significantly in the crude model and borderline in the adjusted models. Few studies have reported the mother’s and daughter’s BD correlation [45, 46]. The study of Maskarinec et al. showed a positive relationship in total breast volume and AFGV between mothers and daughters in late adolescence, but not in %FGV [45]. They suggest that the correlation with %FGV could be apparent only when the total breast maturation has finished due to exogenous exposure, but for AFGV, it could be shown earlier. In our predictive model to determine AFGV 4 years after menarche onset, we also observed that the mother’s AFGV was a variable selected to be included in the model; thus, it might be important to consider it in predictive models for BD.

Although these findings look promising, our study is not exempt of limitations. First, we had a relatively small sample size in some of the Tanner stages. However, to our knowledge, this is the largest study in the literature evaluating BD in peri-pubertal girls. Also, our study sample represents Hispanic low-middle-income girls; thus, our results should be extrapolated with caution to other groups. Thus, the results of our predictive model of %FGV and AFGV at four years after menarche onset cannot be generalizable to other populations. However, our study also has several advantages. We could replicate a low-radiation dose method to measure breast composition in young girls with high precision. Measurements were conducted within an ongoing longitudinal follow-up, allowing us to confirm the validity of the pubertal events, including menarche, and continue the DXA breast measurements in future periods to establish the complete trajectories of AFGV, %FGV, and BV.

In conclusion, this is one of the first studies to explore breast composition before, during, and after puberty in a longitudinal manner using a replicable, low-dose radiation method. We observed that the menarche onset is a critical event for breast development and %FGV and AFGV tracks even at early periods starting in Tanner 4. A better understanding of environmental factors, such as obesity, diet, and endocrine disruptors that have been linked to earlier menarche onset may contribute to comprehending early determinants of BC risk.

### Electronic supplementary material

Below is the link to the electronic supplementary material.

Supplementary Material 1

Supplementary Material 2

Supplementary Material 3

## Data Availability

The data that support the findings of this study are available from the corresponding author, MLG, upon reasonable request.

## Abbreviations

BD: Breast density
BC: Breast cancer
BV: Breast volume
%FGV: %Fibroglandular volume
AFGV: Absolute fibroglandular volume
BV: Breast volume
DXA: Dual-energy X-ray absorptiometry
BMI: Body mass index
GOCS: The Growth and Obesity Chilean Cohort Study
