# Supplementary material for: Breast composition during and after puberty: the Chilean Growth and Obesity Cohort Study
Source: Breast Cancer Res. 2024 Mar 12;26:45. doi: 10.1186/s13058-024-01793-x (PMC10935788; doi:10.1186/s13058-024-01793-x)
Supplement: Supplementary file 1 — Supplementary Material 1 [file 13058_2024_1793_MOESM1_ESM.docx]

**Supplemental Table 1: Descriptive analysis of included and excluded participants. The Growth and Obesity Cohort Study.**

|  | **Included (n= 509)** | **Excluded (n=93)** |  |
| --- | --- | --- | --- |
|  | **Mean (SD)** | **Mean (SD)** | **p-value** |
| Birthweight (grs) | 3.36 (0.39) | 3.36 (0.42) | 0.9652 |
| Weight for age SDS | 0.38 (0.91) | 0.49 (0.94) | 0.3154 |
| Height for age SDS | -0.34(0.93) | -0.21 (0.87) | 0.1985 |
| BMI SDS | 0.86 (0.86) | 0.90 (1.02) | 0.7529 |
| >12 years of maternal education, N (%) | 109 (22.8%) | 5 (23.8%) | 0.910 |

SDS : standard deviation
