# Supplementary material for: Breast composition during and after puberty: the Chilean Growth and Obesity Cohort Study
Source: Breast Cancer Res. 2024 Mar 12;26:45. doi: 10.1186/s13058-024-01793-x (PMC10935788; doi:10.1186/s13058-024-01793-x)
Supplement: Supplementary file 2 — Supplementary Material 2 [file 13058_2024_1793_MOESM2_ESM.docx]

**Supplemental Table 2: %FGV, AFGV, BV and BMI z-score comparison (p-value) across different time points**

|  | **%FGV** | **AFGV** | **BV** | **BMI z score** |
| --- | --- | --- | --- | --- |
| Tanner B1 vs Tanner B2 | 0.258 | 0.189 | 0.094 | 0.298 |
| Tanner B2 vs Tanner B3 | 0.260 | 0.003 | 0.159 | 1.000 |
| Tanner B3 vs Tanner B4 before menarche | 0.080 | 0.132 | 0.987 | 0.472 |
| Tanner B4 before menarche vs Tanner B4 after menarche | <0.001 | <0.001 | <0.001 | 0.319 |
| Tanner B4 before menarche vs TannerB5 before menarche | 0.956 | <0.001 | 0.001 | 0.110 |
| Tanner B4 after menarche vs Tanner B5 after menarche | 0.791 | <0.001 | <0.001 | 0.026 |
| Tanner B5 before menarche vs B5 after menarche | 0.014 | <0.001 | <0.001 | 0.568 |
| Tanner B5 after menarche vs 1 año after menarche | 0.054 | <0.001 | 0.002 | 0.836 |
| 1 year after menarche vs 2 years after menarche | 0.919 | <0.001 | 0.002 | 0.035 |
| 2 years after menarche vs 4 years after menarche | 0.529 | 0.381 | 0.165 | 0.101 |

Data represent p -values of differences according to two-sample Wilcoxon rank-sum test

%FGV: %fibroglandular volumen; AFGV: absolute fibroglandular volume; BV: breast volue; BMI: body mass index
