# Supplementary material for: Breast composition during and after puberty: the Chilean Growth and Obesity Cohort Study
Source: Breast Cancer Res. 2024 Mar 12;26:45. doi: 10.1186/s13058-024-01793-x (PMC10935788; doi:10.1186/s13058-024-01793-x)
Supplement: Supplementary file 3 — Supplementary Material 3 [file 13058_2024_1793_MOESM3_ESM.docx]

**Supplemental Table 3: Determinants of %FGV and AFGV in a fully mature breast at 4 years after menarche onset.**

|  | %FGV | | | | |
| --- | --- | --- | --- | --- | --- |
|  | Crude Model | | Variables selected in Stepwise regression | | |
|  | β | 95%CI | β | 95%CI |  |
| Height z-score | 0.30 | -1.73; 2.33 |  |  |  |
| Mother's %FGV | 0.53 | 0.14; 0.92 |  |  |  |
| Age at DXA | 0.28 | -1.80; 2.36 | -2.11 | -4.02; -0.21 |  |
| BMI z-score | -10.41 | -11.62; -9.19 | -10.27 | -11.71; -8.83 |  |
| Age at Menarche | 1.02 | -0.91; 2.95 |  |  |  |
|  | AFGV | | | | |
|  | Crude Model |  | Variables selected in Stepwise regression | | |
|  | β | 95%CI | β | 95%CI |  |
| Height z-score | 9.95 | -0.73; 20.64 |  |  |  |
| Mother's AFGV | 0.62 | 0.23; 1.00 | 0.58 | 0.19; 0.96 |  |
| Age at DXA | -0.74 | -11.83; 10.35 |  |  |  |
| BMI z-score | 17.2 | 8.64; 25.79 | 19.70 | 9.31; 30.10 |  |
| Age at Menarche | 1.19 | -9.16; 11.54 |  |  |  |

β: beta coefficient, 95%CI: 95% confindence interval,

DXA: Dual-energy X-Ray Absorptiometry, %FGV: % of fibrogladular volume, AFGV: absolute fibroglandular volume; BV: breast volume. BMI: body mass index
